# Fraction Conversion Models Based on Ultrasound Attenuation Coefficient for Assessing Liver Steatosis

**DOI:** 10.3390/diagnostics16071086

**Published:** 2026-04-03

**Authors:** Yin Zhang, Ting Jiang, Chuli Xu, Jiajun He, Hongjun Zhang, Tufeng Chen, Jie Zeng

**Affiliations:** 1 Department of Medical Ultrasound, The Third Affiliated Hospital of Sun Yat-Sen University, Guangzhou 510630, China; zhangy795@mail2.sysu.edu.cn (Y.Z.); xuchli@mail2.sysu.edu.cn (C.X.); zhanghj35@mail.sysu.edu.cn (H.Z.); 2 Department of Radiology, The Third Affiliated Hospital of Sun Yat-Sen University, Guangzhou 510630, China; jiangt5@mail.sysu.edu.cn; 3 Department of Gastrointestinal Surgery, The Third Affiliated Hospital of Sun Yat-Sen University, Guangzhou 510630, China; hejj89@mail2.sysu.edu.cn; 4 Department of Medical Ultrasound, Guangdong Provincial Key Laboratory of Diabetology, Guangzhou Municipal Key Laboratory of Mechanistic and Translational Obesity Research, The Third Affiliated Hospital of Sun Yat-Sen University, Guangzhou 510630, China

**Keywords:** metabolic dysfunction-associated steatotic liver disease, steatosis, magnetic resonance imaging, proton density fat fraction, attenuation coefficient

## Abstract

**Objectives**: We aimed to develop models capable of converting the attenuation coefficient (AC) into a percentage-like index in patients with suspected metabolic dysfunction-associated steatotic liver disease (MASLD). **Methods**: In this retrospective, cross-sectional study, we consecutively enrolled participants with suspected MASLD from the Weight Loss Medical Centre who had undergone both ultrasound examinations that yielded AC results and magnetic resonance imaging (MRI) scans including proton density fat fraction (PDFF). The first model, defined as the PDFF conversion fraction (PCF), used the MRI-PDFF results as the reference standard. The other model, defined as the attenuation level fraction (ALF), converted AC values into percentages based on the range of AC values from 0.5 to 1.0 dB/cm/MHz. Area under the receiver operating characteristic curve (AUC) analysis was used to evaluate the diagnostic performance of the two models. **Results**: Among the 199 participants (mean age, 38.12 ± 9.56 years; 110 male), the PDFF values differed significantly among the different liver segments (*p* < 0.05). The PDFF values of the left liver and right liver were 12.6% and 16.1%, respectively. There was a significant difference between them (*p* < 0.05). The AUCs of the AC, PCF, and ALF were 0.92, 0.93, and 0.87, respectively, for detecting mild steatosis (≥ S1), moderate steatosis (≥S2), and severe steatosis (≥S3) when PDFF values ≥ 5%, ≥15%, and ≥25% were used as the reference standard, respectively. **Conclusions**: The two fraction conversion models (PCF and ALF) yielded good and identical diagnostic accuracies in grading liver steatosis. Considering the heterogeneous pattern of liver steatosis, the ALF was a more objective parameter.

## 1. Introduction

Metabolic dysfunction-associated steatotic liver disease (MASLD) is the most common cause of liver steatosis. Hepatic steatosis, previously termed metabolic dysfunction-associated fatty liver disease (MAFLD) or nonalcoholic fatty liver disease (NAFLD), is the defining feature of MASLD [1]. MASLD has become the most common chronic liver disease worldwide, affecting at least 30% of the general population worldwide [2,3]. Furthermore, MASLD is associated with an increased risk of type 2 diabetes and cardiovascular disease [4,5].

Assessing liver steatosis is important for patients suspected of having MASLD or who have been diagnosed with it. Liver steatosis is defined by the presence of hepatic fat content in 5% of hepatocytes or more [4,6]. Liver biopsy remains the definitive reference standard for steatosis grading. Histologically, grading represents the percentage of hepatocytes containing intracellular lipid-containing vacuoles [4,7]. The Kleiner score is commonly used to grade steatosis [6]. It is based on a four-point classification: steatosis grade 0, steatosis in less than 5% of hepatocytes; 1, 5–33% steatosis; 2, 34–66% steatosis; and 3, more than 66% steatosis. Although liver biopsy is accepted as the most accurate diagnostic method, it is impractical for disease screening and monitoring using liver biopsy as a standard—particularly given the high prevalence of MAFLD—because of its invasiveness, bias toward higher levels of fat, sampling error, and variability in pathologic interpretation [8,9,10].

In addition to liver biopsy, liver fat quantification can also be achieved via magnetic resonance imaging (MRI)-based methods. MRI proton density fat fraction (PDFF) has emerged as an alternative to biopsy. MRI-PDFF can objectively estimate liver fat content with values over the entire range of liver fat content [11,12]. PDFF is expressed as a percentage (range, 0–100%) and defined as PDFF = F/(W + F), where F and W are the unconfounded signals from protons within mobile triglycerides and mobile water molecules, respectively [11,13]. MRI-PDFF has been widely used as a non-invasive alternative reference method for quantifying liver steatosis and monitoring changes over time in many diagnostic studies and clinical trials [14,15].

Ultrasound (US) techniques for assessing liver steatosis are inexpensive and widely available, making them more suitable for population-level diagnosis and risk stratification, considering the high prevalence of MAFLD. The attenuation coefficient (AC) used for estimating liver steatosis has been widely studied [15,16]. The AC is the rate of amplitude loss of the US beam as it travels through tissue and propagates through the tissue, quantified in decibels per unit depth (in centimeters). It is frequency dependent. Thus, the AC has a unit of dB/cm/MHz. Higher attenuation values are present in liver tissues with more severe steatosis. Many studies have been published regarding diagnostic accuracy in assessing liver steatosis [14,17]. As mentioned above, AC results are expressed in dB/cm/MHz, which is a long unit, and it is not as easy to understand as the unit of percentage in PDFF and liver biopsy results. Researchers have recommended developing models for converting AC results into a percentage-like index anchored to MRI-PDFF [14]. The US-derived fat fraction value (Siemens Healthineers), which combines attenuation and the backscatter coefficient, was first reported as a percentage-like index anchored to MRI-PDFF [18,19].

Therefore, in this study, we aimed to develop models with which to convert AC results into a percentage-like index. The first model used the MRI-PDFF as the reference standard. The results of this model were defined as the PDFF conversion fraction (PCF). Because a heterogeneous pattern of steatosis has been reported in up to 60% of patients with NAFLD [20], the other model converted the AC value into a percentage based on the range of AC values from 0.5 to 1.0 dB/cm/MHz. The results obtained from the second model were defined as the attenuation level fraction (ALF). Furthermore, we compared the diagnostic performance of the two models.

## 2. Materials and Methods

### 2.1. Study Participants

We conducted a retrospective, cross-sectional, diagnostic, single-site study. It was approved by the institutional review board, and the requirement for obtaining informed consent from patients was waived (approval number: GR2024-108-02). Participants from the Weight Loss Medical Center who had both undergone US examinations with AC results and MRI scans with PDFF data were consecutively enrolled between September 2021 and May 2024. The inclusion criteria were being between 18 and 80 years old, being a Chinese citizen, and having an interval between US and MRI examinations of no more than one week. The exclusion criteria were being pregnant; having undergone systemic chemotherapy or biological therapy; being afflicted by tumors in the liver lobe segment to be sampled; and having failed or unreliable US or MRI-PDFF examination. Patients’ medical histories and anthropometric and biochemical data were recorded.

### 2.2. AC Measurement Procedures

A radiologist (J. Zeng) with more than 10 years of experience in liver US examination independently performed the AC measurements. The radiologist was blinded to the patient’s clinical information and MRI results. The AC measurements were performed via an Aplio i900 US system (Canon Medical Systems Corporation, Otawara, Japan) with an i8CX1 convex probe (frequency range, 1–8 MHz) or a Resona R9 US system (Mindray Bio-Medical Electronics Co., Ltd., Shenzhen, China) with a SC6-1U convex probe (frequency range, 1–6 MHz). Before ultrasound examination, patients fasted for at least 6 h. Each patient was placed in the supine position with his or her right arm in maximal abduction. The probe was positioned in the intercostal spaces of the right lobe of the liver. The operator positioned the target area of the liver under the guidance of a conventional, real-time B-mode image. When the target area was located, patients were asked to stop breathing for approximately 3 s. The region of interest (ROI) for the AC measurement was placed at least 1 cm below the liver capsule to avoid producing reverberation artifacts and inside liver parenchyma. A movable ROI was taken. It was placed close to the center of the imaging area, avoiding intrahepatic ductal structures. The dimensions of the ROI box were 2 × 2 cm in the Mindray device or approximately 3 × 4 cm in the Canon device. The reliability of the results was displayed in terms of the R2 value for the Canon device or the credibility index for the Mindray device . The AC values with R2 values or credibility indices greater than or equal to 0.80 were considered valid data, as per information provided by the manufacturers. The AC values were reported in dB/cm/MHz. Imaging examinations were performed until five valid AC values had been obtained, and the median value was used for the statistical analysis. The results were considered unreliable when AC values with an IQR-to-median ratio greater than 30%.

### 2.3. MRI-Derived Proton Density Fat Fraction

MRI was performed via a 3.0T MRI scanner (Discovery 750, GE Healthcare, Milwaukee, WI, USA). Axial breath-hold IDEAL-IQ sequences were acquired with the following parameter settings: TR = 4.0 ms, section thickness = 5.0 mm, bandwidth = 125 kHz, FOV = 42 cm × 42 cm, matrix = 256 × 256, flip angle = 3°, and NEX = 1.00. The maximum echo of the TE was 5.5 ms, and the minimum echo was 1 ms. The ROI with the maximum fit (avoiding major blood vessels, bile ducts, artifacts, lesions, other organs, and the liver margin) was manually placed on the source images of each liver segment. The ROI was converted into a parametric PDFF map to obtain the PDFF values of each liver segment. The whole-liver PDFF value was the average of the PDFF values of nine liver segments, with the IV segment further subdivided into IVa and IVb. The left liver PDFF value was the average of the PDFF values of segments I, II, III, IVa, and IVb, and the right liver PDFF value was the average of the PDFF values of segments V, VI, VII, and VIII. The grades of mild steatosis (S ≥ 1), moderate steatosis (S ≥ 2), and severe steatosis (S ≥ 3) were defined as PDFF ≥ 5%, ≥15%, and ≥25%, respectively.

### 2.4. Statistical Analysis

Continuous variables are expressed as means ± standard deviations or medians with interquartile ranges (IQRs), whereas categorical variables are presented as counts and percentages. The Kolmogorov–Smirnov test or D’Agostino–Pearson test was used to assess the normality of the MRI-PDFF and AC values. The Kruskal–Wallis H test was applied to evaluate differences in the distributions of ultrasound parameters across different PDFF grades. Post hoc tests further confirmed the differences between the groups. The bootstrap method for independent samples *t*-test was used to compare AC measurements between the two ultrasound devices. Pearson correlation coefficients were used to assess the linear relationship between AC values and MRI-PDFF, and a regression equation was derived accordingly.

Receiver operating characteristic (ROC) curve analysis with 95% confidence intervals (CIs) was used to evaluate the diagnostic performance of each parameter. The DeLong test was used to compare the areas under the ROC curves (AUCs) among different models [21]. The AUC values were considered excellent, good, and fair at 0.9–1.0, 0.8–0.9, and 0.7–0.8, respectively [22]. The optimal cut-off values for diagnosis were determined based on the highest Youden’s index value [23]. All the statistical analyses were performed via SPSS Statistics (version 25.0), R Studio (version 4.3.1), and MedCalc Software (version 11.2). All the statistical tests were two-sided; the significance level was 0.05 for statistical inference.

## 3. Results

### 3.1. Participant Characteristics

**Figure 1:**
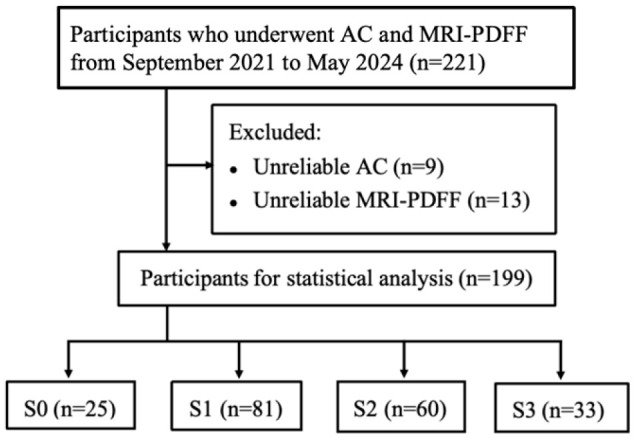
Flowchart of the study population. AC = attenuation coefficient, MRI-PDFF = magnetic resonance imaging proton density fat fraction, S0 = no steatosis, S1 = mild steatosis, S2 = moderate steatosis, and S3 = severe steatosis.

A total of 221 participants with suspected MASLD who had undergone US with AC results and MRI-PDFF from September 2021 to May 2024 were consecutively enrolled in our study. A total of nine (4%) participants with unreliable AC results (an IQR-to-median ratio > 30%) and 13 (6%) participants with unreliable MRI-PDFF data (respiratory artifacts) were excluded, and the remaining 199 participants were included in the study (Figure 1). Among the 199 participants, the ACs of 115 participants were measured using a Mindray ultrasound device, whereas the ACs of 84 participants were measured via a Canon ultrasound device. The participants were divided into the S0 group (*n* = 25), S1 group (*n* = 81), S2 group (*n* = 60), and S3 group (*n* = 33) according to PDFF cut-off values of 5%, 15%, and 25%. The baseline demographic characteristics of the participants and biochemical, MRI-PDFF, and AC findings are summarized in Table 1.

### 3.2. Analysis of the PDFF Values in Different Liver Segments

**Figure 2:**
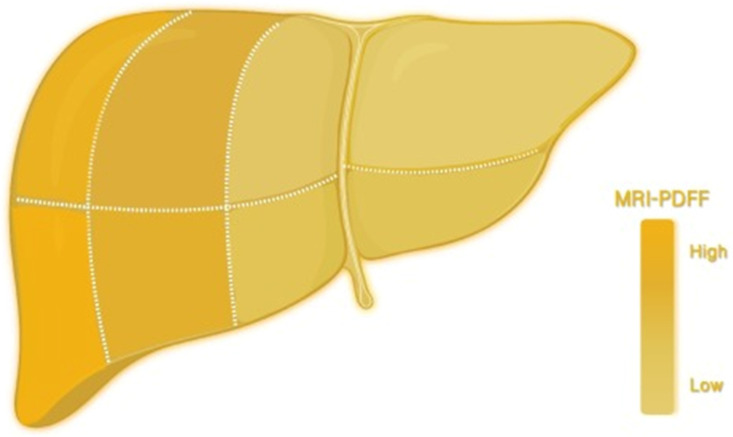
The pattern diagram shows MRI-PDFF values across different segments of the liver. Color represents the magnitude of PDFF values. MRI-PDFF = magnetic resonance imaging proton density fat fraction.

**Figure 3:**
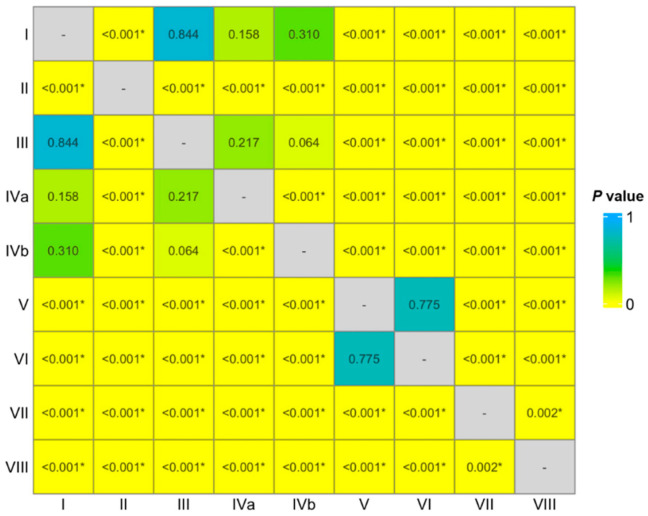
Heatmap presenting the results of a pairwise comparison across different segments of the liver. * indicates a statistically significant difference (*p* < 0.05).

The median PDFF values for liver segments I, II, III, IVa, IVb, V, VI, VII, and VIII were 13.5%, 11.4%, 13.1%, 12.1%, 13.0%, 15.1%, 15.7%, 16.6%, and 16.4%, respectively. The PDFF values differed significantly among the different liver segments (*p* < 0.05) (Figure 2). The post hoc pairwise comparisons of the PDFF values among the different liver segments are presented in Figure 3. The PDFF values of the left and right segments of the liver were 12.6% and 16.1%, respectively. In addition, there was a significant difference between them (*p* < 0.05).

### 3.3. Analysis of AC Values Between Two Devices

Among the 25 S0 patients, the ACs of 18 (72%) were measured using the Canon device, while the ACs of seven (28%) were measured using the Mindray device. Among the 81 S1 patients, the ACs of 33 (41%) and 48 (59%) individuals were measured using the Canon and Mindray devices, respectively. Among the 60 S2 patients, the ACs of 21 (35%) and 39 (65%) individuals were measured using the Canon and Mindray devices, respectively. Finally, among the 33 S3 patients, the ACs of 12 (36%) and 21 (64%) individuals were measured using the Canon and Mindray devices, respectively. The difference in the mean AC value obtained using the Mindray or Canon device was 0.01 at the S2 or S3 grade. The largest difference in the mean AC values obtained via the Mindray or Canon device was 0.04 at the S0 grade. According to the bootstrap method for the independent-samples *t*-test, there were no statistically significant differences in the AC measurements between Canon and Mindray across the four distinct fat steatosis grades (all *p* > 0.05) (Table 2).

### 3.4. Model of the PDFF Conversion Fraction (PCF)

The MRI-PDFF values deviated from normality (*p* < 0.05). Square-root transformation was applied to improve data symmetry and meet the assumptions of linear regression analysis, after which the $\sqrt{MRI-PDFF}$ values were normally distributed (*p* > 0.05). A comparison of the MRI-PDFF and AC in the four groups is summarized in Table 3. As the steatosis group increased, its mean AC value increased. The differences in the MRI-PDFF and AC results were statistically significant between adjacent groups (*p* < 0.05).

**Figure 4:**
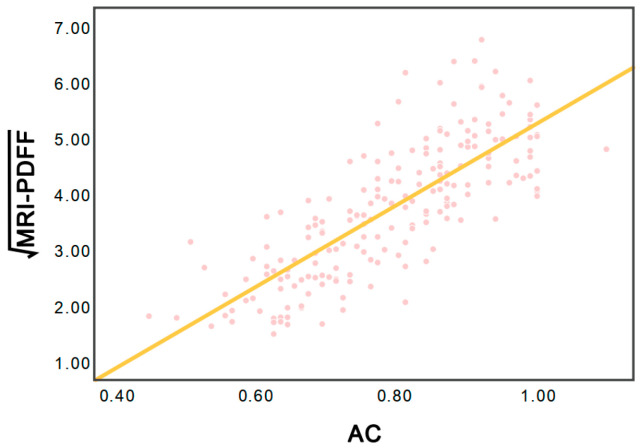
Scatterplot showing the relationship between MRI-PDFF and AC. AC = attenuation coefficient, and MRI-PDFF = magnetic resonance imaging proton density fat fraction.

The AC value revealed a linear correlation with $\sqrt{MRI-PDFF}$ (r = 0.77; *p* < 0.05), yielding the following conversion formula: $\sqrt{MRI-PDFF}$ = 7.21 × AC − 1.95 (Figure 4). To maintain the percentage-based nature of the MRI-PDFF, the PDFF conversion fraction (PCF) model was established as follows: PCF (%) = 0.1 × $\sqrt{MRI-PDFF}$ × 100% = (0.721 × AC − 0.195) × 100%.

### 3.5. Model of the Attenuation Level Fraction (ALF)

In this study, among the 199 participants, the AC values were less than 0.5 dB/cm/MHz in two individuals (namely, 0.48 and 0.44 dB/cm/MHz) and exceeded 1.0 dB/cm/MHz in one individual, with a value of 1.1 dB/cm/MHz. The remaining 196 participants had AC values within the range of 0.5–1.0 dB/cm/MHz, and these values were normally distributed (*p* > 0.05).

An attenuation level fraction (ALF) model was constructed by linearly mapping AC values between 0.5 and 1.0 dB/cm/MHz to a 0–100% scale, employing the following conversion formula: ALF (%) = (AC − 0.5)/(1.0 − 0.5) × 100%. Values below 0.5 dB/cm/MHz were assigned a minimum threshold of 0.5 dB/cm/MHz and mapped to 0%, whereas values above 1.0 dB/cm/MHz were assigned a maximum threshold of 1.0 dB/cm/MHz and mapped to 100%.

### 3.6. Diagnostic Performances of the AC, ALF, and PCF

The AUCs and the diagnostic performance of the model’s cut-off values are detailed in Table 4. At cut-off values of 0.73 for AC, 45% for ALF, and 33% for PCF, the identification of ≥S1 yielded identical AUC values of 0.92 (95% CI, 0.87–0.96). At cut-off values of 0.77 for AC, 53% for ALF, and 36% for PCF, the identification of ≥S2 yielded identical AUC values of 0.93 (95% CI, 0.90–0.96). At cut-off values of 0.86 for AC, 71% for ALF, and 42% for PCF, the identification of ≥S3 yielded identical AUC values of 0.87 (95% CI, 0.82–0.92).

## 4. Discussion

The liver biopsy and MRI-PDFF results are expressed as percentages. However, AC values are expressed in dB/cm/MHz. To improve its clinical use, researchers have recommended developing models with which to convert results into a percentage-like index anchored to MRI-PDFF [14]. In our study, we developed a model for PCF. The AUCs of the PCF in detecting mild steatosis (S ≥ 1), moderate steatosis (S ≥ 2), and severe steatosis (S ≥ 3) were 0.92, 0.93, and 0.87, respectively. The MRI-PDFF results are sometimes reported as a range of factions in clinical practice due to the heterogeneous pattern of steatosis. Therefore, we also developed a metric that did not rely on MRI-PDFF results, which was defined as ALF. The diagnostic performances of AC, PCF, and ALF in detecting different steatosis grades were the same in terms of AUCs, specificity, and sensitivity. Diagnostic performance remained unchanged after the linear transformation of the AC values. Indeed, the cut-offs for AC, PCF, and ALF were different.

A nonuniform distribution of fat in the liver is common. In our study, the median PDFF value among the participants was lowest in liver segment II (11.4%). The highest PDFF value was 16.6%, observed for liver segment VII. The PDFF values of the left and right segments of the liver were 12.6% and 16.1%, respectively. The PDFF values differed significantly among the different liver segments. The whole-liver PDFF value is the average of the PDFF values of nine liver segments. The MRI-PDFF results might not precisely reflect the fat content of the liver. Alternatively, the fat content of the liver may not inherently be a precise value in some cases. The MRI-PDFF can measure nine liver segments. However, the AC is usually measured at the same location in the right lobe of the liver. As the methods for measuring the AC and MRI-PDFF are completely different, the use of the MRI-PDFF as a reference for converting the AC value into a percentage-like index anchored to MRI-PDFF might result in errors.

Because a heterogeneous pattern of steatosis has been reported in up to 60% of patients with NAFLD, the placement of a single ROI is unlikely to be sufficient for correctly estimating the true severity of liver fat [20]. Generally, it is recommended that multiple, large ROI should be placed at multiple representative locations across the liver [20]. The study by Campo et al. indicated that clinicians and researchers should sample as much of the liver as possible using multiple large ROIs [24]. Although the World Federation for Ultrasound in Medicine and Biology (WFUMB) guidance recommends performing AC measurements with three to five acquisitions at the same location in the right lobe of the liver [15]. These data suggest that future studies should evaluate whether multi-segment AC sampling improves agreement with whole-liver MRI-PDFF. Liver biopsy is still considered the reference standard for steatosis grading. However, liver biopsy is invasive and poses a risk of significant complications, limiting the number of sampling attempts. Additionally, the histologic specimen taken represents only a tiny fraction (1/50,000) of the entire liver, making it unlikely to be representative of features that are heterogeneously distributed.

In our study, some patients were assessed using the Canon device, while the others were assessed using the Mindray device. We compared the AC values obtained using the Canon or Mindray device. The difference in the mean AC values obtained via Mindray or Canon was only 0.01 at grades S2 and S3. There were no statistically significant differences in the AC measurements between Canon and Mindray across the four distinct fat steatosis grades. Ferraioli et al. reported substantial variability in AC values obtained with different US systems, precluding interchangeability between systems for liver steatosis diagnosis and follow-up imaging [25]. In our study, the AC values obtained using the Canon or Mindray device were not compared head by head, but instead only in the same steatosis grade. However, considering the heterogeneous pattern of steatosis and the variability in intrahepatic fat among individuals, we must also consider how much of a difference in measured values between devices is acceptable. In the study by Ferraioli et al., eight US systems were used [25]. Patients with fatty liver need to be continuously followed up to monitor changes in the degree of fat. The non-interchangeability between measuring devices can cause great inconveniences in clinics, and adopting appropriate methods to make measured AC values comparable between devices is an important issue. The development of standardized phantoms that vendors can use to standardize their measurements may be a feasible method. In our study, no statistically significant differences were observed between the AC measurements obtained using the Canon or Mindray device. Therefore, we did not calculate Mindray or Canon values separately in the final statistics.

The US-derived fat fraction (UDFF) value (Siemens Healthineers), which combines attenuation and the backscatter coefficient, is reported as a percentage [26]. It is the first US technique used to determine a percentage-like index anchored to MRI-PDFF. Labyed and Milkowski reported that the AUCs of UDFF were 0.94, 0.88, and 0.83 for histologic steatosis of grade 1 or greater, 2 or greater, and 3, respectively [18]. In the study by Huang et al., which was conducted using PDFF ≥ 5%, ≥15%, and ≥25% as the reference standard for detecting mild, moderate, and severe hepatic steatosis, the best cut-off values of UDFF were 7.6% (AUC = 0.90), 15.9% (AUC = 0.90), and 22.3% (AUC = 0.91), respectively [27]. In our study, PCF was converted from AC, not combined with the backscatter coefficient, using the MRI-PDFF as the reference standard. Steatosis was also graded via the same PDFF cut-offs as those used in our study. The AUCs of PCF were 0.92, 0.93, and 0.87, respectively. These results are similar to those reported in previous studies. The best cut-off values for PCF were 33%, 36%, and 42%, which are different from those reported in the previous study [27]. Although MR was used as the reference standard in both our study and previous studies, the diagnostic thresholds could not be the same as those for MR. Furthermore, the PDFF values deviated from normality in the sample in our study. We performed a square-root transformation on the PDFF values, and the transformed values conformed to a normal distribution. Therefore, the results obtained by the model using PDFF as the reference standard cannot be precisely equivalent to the MR-measured values. In addition, the non-uniform distribution of fatty liver may lead to larger errors.

The ALF does not depend on the MRI results and represents the position of the AC measurement within the range of AC values, where 0 is the minimum and 1 is the maximum. The range of AC values used in our study was 0.5–1.0 dB/cm/MHz. A previous study reported that the AC values of 95% of patients fall within the range of 0.5–1.1 dB/cm/MHz [14]. Nevertheless, since the upper limit of the AC measurement value for the Mindray equipment is 1.0 dB/cm/MHz, we chose a range of 0.5–1.0 dB/cm/MHz. Among the 199 participants in our study, the AC values were less than 0.5 dB/cm/MHz for only two individuals and exceeded 1.0 dB/cm/MHz for only one individual when assessed via the Canon device. AC values below 0.5 or exceeding 1.0 were assigned values of 0.5 and 1.0, respectively. The diagnostic performance of the AC, PCF, and ALF in detecting different steatosis grades was the same in terms of AUCs, specificity, and sensitivity. These results indicate that linear transformations did not change the AUCs, but the cut-offs were different. The best cut-off values of the ALF were 45%, 53%, and 71% for detecting mild, moderate, and severe steatosis, respectively.

Our study has certain limitations. First, our sample consisted of a variable distribution of participants with a variety of different steatosis grades, particularly S0. Second, AC values were obtained using one of two devices: Canon or Mindray. We did not calculate Mindray or Canon values separately in the final statistics. Third, the range of AC values used in our study was 0.5–1.0 dB/cm/MHz. As mentioned earlier, a previous study reported that the AC values of 95% of patients fall within the range of 0.5–1.1 dB/cm/MHz [14]. Only three of the 199 participants had AC measurements outside this range in our study. Fourth, AC values with an IQR-to-median ratio greater than 30% were excluded from our study. The reliability criterion according to the WFUMB’s guidance on liver fat quantification suggests that an IQR-to-median ratio should be no more than 15%. However, the diagnostic performance of our study was similar to that of previous studies [18,27]. Fifth, our study cohort included participants with a mean BMI of 33.7 kg/m^2^. The developed models (PCF/ALF) are not necessarily suitable for non-obese patients with lean MASLD. Sixth, there was no external validation cohort.

## 5. Conclusions

In conclusion, the two fraction conversion models (PCF or ALF), which used the MRI-PDFF as the reference standard or were based on the range of AC values, achieved good and identical diagnostic accuracies in grading liver steatosis. Considering the heterogeneous pattern of steatosis, the ALF was a more objective parameter. Prospective validation studies are required.

## Figures and Tables

**Table 1:** The participants’ baseline demographic, biochemical, MRI-PDFF, and AC results.

Characteristics	Value	Number
Age (y)	31 ± 8	199
Sex *		199
M	84 (42%)	
F	115 (58%)	
BMI (kg/m^2^)	33.7 ± 4.5	192
Waist (cm)	105.2 ± 15.7	193
Hypertension *	48 (27%)	177
Fasting Blood Glucose (mmol/L)	5.41 ± 1.62	185
Oral Glucose Tolerance Test-2 h (mmol/L)	8.22 ± 3.20	175
HbA1c (%)	5.7 ± 1.1	168
Triglycerides (mmol/L)	2.06 ± 1.79	171
High-Density Lipoprotein Cholesterol (mmol/L)	1.09 ± 0.38	171
MRI-PDFF *		199
S0 (<5%)	25 (13%)	
S1 (5≤ to <15%)	81 (41%)	
S2 (15≤ to <25%)	60 (30%)	
S3 (≥25%)	33 (17%)	
AC (dB/cm/MHz)	0.79 ± 0.13	199

Note: Unless otherwise indicated, data are presented as mean ± standard deviation (SD). * Data are presented as number of participants, with percentages in parentheses. AC = attenuation coefficient, MRI-PDFF = magnetic resonance imaging proton density fat fraction, S0 = no steatosis, S1 = mild steatosis, S2 = moderate steatosis, and S3 = severe steatosis.

**Table 2:** AC values of different steatosis grades obtained using the Mindray or Canon device.

Steatosis Grade	Canon	Mindray	*p* Value *
S0	0.63 ± 0.06	0.59 ± 0.12	0.33 (−0.04, 0.11)
S1	0.74 ± 0.10	0.71 ± 0.08	0.06 (0.00, 0.08)
S2	0.88 ± 0.10	0.87 ± 0.08	0.68 (−0.04, 0.06)
S3	0.92 ± 0.05	0.93 ± 0.07	0.78 (−0.04, 0.03)

Note: Unless otherwise indicated, data are presented as mean ± standard deviation (SD). * Data in parentheses are 95% CIs. AC = attenuation coefficient.

**Table 3:** MRI-PDFF and AC values for various steatosis groups.

Parameters	S0 (*n* = 25)	S1 (*n* = 81)	*p* * Value	S2 (*n* = 60)	*p* ** Value	S3 (*n* = 33)	*p* *** Value
MRI-PDFF	3.5% ± 0.7%	9.6% (7.0–12.5%) ^a^	<0.05	19.4% ± 3.0%	<0.05	28.4% (26.3–35.1%) ^a^	<0.05
AC	0.62 ± 0.08	0.73 ± 0.09	<0.05	0.87 ± 0.09	<0.05	0.92 ± 0.06	<0.05

Note: Unless otherwise indicated, data are presented as mean ± standard deviation (SD). ^a^ Data are medians, with IQRs in parentheses. *p* *: compared with S0; *p* **: compared with Sl; *p* ***: compared with S2. S0 = no steatosis, S1 = mild steatosis, S2 = moderate steatosis, and S3 = severe steatosis.

**Table 4:** Performance of various parameters for discrimination of various steatosis grades.

		AUC *	Cut-Off	Sensitivity *	Specificity *
AC					
	≥S1	0.92 (0.87, 0.96)	0.73	76% (69%, 82%)	96% (80%, 100%)
	≥S2	0.93 (0.90, 0.96)	0.77	95% (88%, 98%)	77% (67%, 84%)
	≥S3	0.87 (0.82, 0.92)	0.86	91% (76%, 98%)	74% (67%, 81%)
ALF					
	≥S1	0.92 (0.87, 0.96)	45%	76% (69%, 82%)	96% (80%, 100%)
	≥S2	0.93 (0.90, 0.96)	53%	95% (88%, 98%)	77% (67%, 84%)
	≥S3	0.87 (0.82, 0.92)	71%	91% (76%, 98%)	74% (67%, 81%)
PCF					
	≥S1	0.92 (0.87, 0.96)	33%	76% (69%, 82%)	96% (80%, 100%)
	≥S2	0.93 (0.90, 0.96)	36%	95% (88%, 98%)	77% (67%, 84%)
	≥S3	0.87 (0.82, 0.92)	42%	91% (76%, 98%)	74% (67%, 81%)

* Data in parentheses are 95% CIs. AC = attenuation coefficient, ALF = attenuation level fraction, PCF = PDFF conversion fraction, S1 = mild steatosis, S2 = moderate steatosis, and S3 = severe steatosis.

## Data Availability

The datasets generated and/or analyzed during the current study are not publicly available due to concerns regarding patient privacy, but are available from the corresponding authors upon reasonable request.
